# SAVI Space—combinatorial encoding of the billion-size synthetically accessible virtual inventory

**DOI:** 10.1038/s41597-025-05384-z

**Published:** 2025-06-23

**Authors:** Malte Korn, Philip Judson, Raphael Klein, Christian Lemmen, Marc C. Nicklaus, Matthias Rarey

**Affiliations:** 1 University of Hamburg, ZBH – Center for Bioinformatics, 22761 Hamburg, Germany; 2 Heather Lea, Bland Hill, Norwood, Harrogate, HG3 1TE England; 3 BioSolveIT GmbH, St. Augustin, Sankt Augustin, Germany; 4 NCI, NIH, CADD Group, NCI-Frederick, Frederick, Maryland, 21702 USA

**Keywords:** Virtual screening, Data processing

## Abstract

The Synthetically Accessible Virtual Inventory (SAVI) comprises a huge molecule collection. LHASA transform rules, originally intended for retro-synthetic analysis, were applied to Enamine Building Blocks in a forward synthetic manner. Adding new transforms, expressly developed for SAVI, resulted in SAVI-Lib-2020, a collection of more than a billion synthetically accessible compounds. Handling a billion molecules explicitly is computationally quite demanding for drug discovery applications. SAVI-Space-2024 was created to address this shortcoming. In this paper, we describe the design and implementation of SAVI-Space-2024. We emphasize its reaction-driven combinatorial data structure that encodes transformation rules as reaction SMARTS and applies them in a combinatorial manner. Based on Enamine Building Blocks, this approach yields 7.5 billion molecules while requiring only a fraction of the memory (1.4 GB compared to 210 GB). Furthermore, the improved search capabilities — including fast similarity and substructure searches and docking applications on standard hardware — represent a significant advance over the enumerated SAVI library.

## Background & Summary

A huge number of small organic molecules are available as starting points for early-phase drug discovery. Since standard virtual screening technology handles compounds one by one, it scales linearly with the number of compounds to consider. This process becomes more and more challenging and expensive with ever-growing compound sets. In an attempt to address this challenge, the concept of chemical fragment spaces was invented^1,2^. Today, make-on-demand offerings like the Enamine REAL Space^3^ or the eMolecules eXplore Space^4^ as well as large in-house collections at pharmaceutical companies are frequently stored in this manner. See recent reviews on chemical spaces^5–7^ for details. Chemical fragment spaces provide a memory-efficient alternative to storing enumerated products from large compound collections. The basic idea behind such spaces is the conversion of chemical reactions into connection rules, which are stored alongside large numbers of preprocessed reactants — so-called fragments or synthons. Besides storage efficiency, chemical fragment spaces address the need for fast searching by similarity, substructure, or even three-dimensional features. Tools like Feature Trees for fuzzy 2D pharmacophore mapping^1^, SpaceLight for fingerprint-based similarity search^8^, and SpaceMACS for substructure search^9^, can search and analyze chemical fragment spaces in seconds to minutes at most on standard desktop computers. Following similar concepts to store fragments and rules rather than enumerated compound libraries led to alternative solutions with similarly impressive results even in 3D^10–14^. The Synthetically Accessible Virtual Inventory (SAVI, for clarity, the SAVI-Lib-2020) is a collection of more than a billion small organic compounds created by applying reaction patterns to building blocks^15,16^. In a nutshell, the concept behind SAVI is based on a customized and adapted selection of LHASA transform rules along with newly created transforms and combined with commercially available building blocks^17^. The LHASA transform rules were developed by organic chemists between the 1970s and 1990s and further extended for the development of SAVI^18–20^. These rules describe robust reactions, so the products of SAVI are likely to be synthetically accessible. The LHASA transforms, used for the generation of the SAVI-Lib-2020, can be seen in Table S1. At present, SAVI is available only as an enumerated list of reaction products (the SAVI-Lib-2020), which makes the collection time-consuming to explore and analyze, because of its sheer size. The SAVI-Lib-2020 was created with the CACTVS toolkit, a collection of programs for cheminformatics tasks^21,22^. The CACTVS toolkit can read and apply the LHASA transform rules, written in the CHMTRN/PATRAN language^23^. For each reaction in the LHASA transform rules, there is a script-like document that contains the LHASA transform pattern, so-called KILL and SCORE statements (specifically expressed in ADD or SUBTRACT CHMTRN clauses, assigning score increments or decrements, respectively). Also references to the original literature, the organic chemist who wrote the reaction transform rule, and ratings of the reaction conditions are included. While a transform encodes how a compound is formed based on specific building blocks, KILL statements check for substructures that might lead to side product formation or inhibit the reaction altogether. SCORE statements further rate the reactions concerning the complexity of reaction conditions and the expected yield. The LHASA transform pattern strings are similar to reaction SMARTS or SMIRKS, originally developed by Daylight Information Systems^24^, which is today a quasi-standard for chemical patterns. Almost all elements of the LHASA transform pattern strings can be directly translated to SMARTS expressions. In the CHMTRN/PATRAN language, there are atom properties, which describe the presence of the atom in a particular environment, e.g. a functional group. In SMARTS, this is possible to implement by using recursive SMARTS pattern. An advantage of the LHASA transform rules are additional bond properties, besides the bond type. For example, the fusion bond property is neither available in SMARTS nor in reaction SMARTS. As shown below, the fusion bond property is one of the few aspects from CHMTRN/PATRAN that can be mapped only approximately to SMARTS. The SCORE and KILL statements used in addition to LHASA transform rules rate specific reactions or filter out unstable or sterically hindered products^15^. Both types of statements are written in a natural language-inspired programming language. Like in most programming languages, the CHMTRN/PATRAN language contains the concept of conditional statements. In the workflow used to generate the SAVI-Lib-2020, all SCORE and KILL statements are applied individually to every single product assembled with the transform rules. When a product triggers a KILL statement, this product is filtered out and not saved. The SCORE statements are used to evaluate the reaction outcomes in terms of stereochemistry and reliability and are related to the expected yield. However, these were not considered for the current version of the SAVI-Space (SAVI-Space-2024). In contrast to the methods outlined above, chemical fragment spaces exploit the combinatorial nature of large compound collections by storing the essence of reaction rules and pre-processed reactants. Thus, compounds are stored implicitly only. Reactants are converted to synthons containing dummy atoms as linkers with specific types such that the reaction can be easily encoded by link type pairs and some additional data describing required local changes. In a two-component reaction with reactants A and B, this format requires that every available reactant of type A is expected to react with every reactant of type B. Therefore, it is not possible to define exceptions to individual products. Instead, it is necessary to apply any constraints during reactant preprocessing already, e.g. by applying some fine grain filtering or by defining multiple copies of the same reaction rule for different sub-sets of reagents.

## Methods

First, the methodology used to create a fragment space based on the SAVI definitions and rules is explained. For reasons of clarity and consistency, this newly created fragment space is called SAVI-Space in the following. There are three versions of SAVI-Space, which differ in the building blocks and the exact chemical model that is implemented. All three versions, as well as the enumerated SAVI-Lib-2020, are listed with some key characteristics in Table 1. When generating SAVI-Space every LHASA transform rule describing one reaction scheme is processed independently. First, the LHASA transform pattern string is translated to reaction SMARTS and used to filter the building blocks for matching reactants. Then the KILL statements are translated to a collection of SMARTS and applied to these filtered reactants.

**Table 1:** Different handling of the LHASA transform rules.

Handling of	SAVI-Lib-2020	SAVI-Space-2020 (Lib-2020 rules)	SAVI-Space-2020	SAVI-Space-2024
Aromaticity model	CACTVS	CACTVS	NAOMI	NAOMI
Functional groups	can be aromatic	can be aromatic	must not be aromatic*	must not be aromatic*
Matching duplicates	unify matches with the same atoms	unify matches with the same atoms	based on possible product mixtures**	based on possible product mixtures**
Building Blocks Collection (Enamine)	12 / 2020	12 / 2020	12 / 2020	07 / 2024

* unless specified otherwise; ** stereoisomers are ignored.

### Translating the LHASA transform pattern

**Fig. 1:**
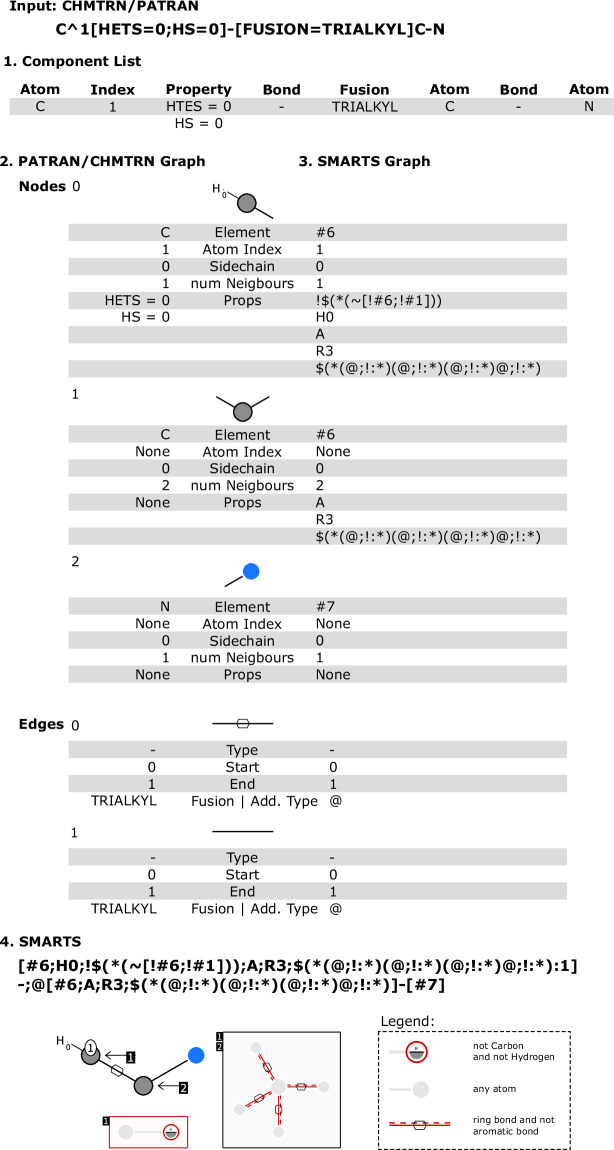
The step-by-step conversion of a CHMTRN/PATRAN string into a SMARTS pattern. The input CHMTRN/PATRAN string is first broken down into a component list (1) that classifies each segment of the pattern. The property “HETS” indicates the number of neighboring hetero atoms, the property “HS” indicates the number of bonded hydrogens, and the “-” indicates a single bond. This list is then used to create a graph representation of the CHMTRN/PATRAN structure (2). Next, the corresponding SMARTS graph (3) is created using the translation mapping. The additional type “@” of the edge is given by the bond property “TRIALKYL” and indicates that it is a ring bond. Finally, a SMARTS string is created by iterating through the node and edge list in the output (4). The pattern is shown schematically in the lower part. (Image created with SMARTSview^51^).

Because the LHASA transform rules and the reaction SMARTS syntax have similar semantics, the translation of the LHASA transform rules is reasonably straightforward. To semi-automate the process, we implemented a transpiler translating the LHASA transform patterns into reaction SMARTS patterns. The code of the transpiler is available on GitHub (https://github.com/rareylab/SAVI-Space). See Fig. 1 for an example translation.

The CHMTRN/PATRAN language consists of several categories of terminologies. These include atom, atom properties, atom mapping, functional groups, bonds, and bond properties. For each of these categories, a comprehensive mapping was established. This mapping delineates each LHASA transform keyword and its corresponding SMARTS expression, and builds the key part of the transpiler. The complete mapping developed can be found in Table S6–S14.

All functional groups covered in SAVI are described in the supplementary material of the publication of Judson *et al*.^23^ Care was taken to convert functional groups to SMARTS with identical semantics including implicit exclusions. For example, bonds of functional groups must not be part of an aromatic ring system, unless explicitly specified. When evaluating chemical reactions, the electronic properties of the functional groups are particularly important. These are influenced and changed if functional groups are part of aromatic systems. This distinction is not always made in the SAVI-Lib-2020. The rules used when creating the chemical spaces and the source of the building block data are the two main components that combine to make the various variants of the SAVI-Space. The most recent version, “SAVI-Space-2024”, adheres to the updated rules to guarantee high synthetic feasibility and reagent availability. It was created using the Enamine Building Blocks from 2024. In an attempt to mimic the chemical semantics of the SAVI-Lib-2020, adjustments were made to the translated pattern and the chemistry model, especially concerning aromaticity handling (Lib-2020 rules). The “SAVI-Space-2020(Lib-2020 rules)” uses the same Enamine Building Blocks as the SAVI-Lib-2020 and was specifically created to evaluate how well the Lib-2020 rules have been mimicked. This evaluation is performed by comparing the products of the SAVI-Space-2020(Lib-2020 rules) with those in the SAVI-Lib-2020. Additionally, another version, “SAVI-Space-2020”, applies the updated rules to the 2020 building blocks, offering an alternative perspective on the chemical space. An overview of the different handling of the rules is shown in Table 1.

To map atoms between reactants and products an approach like the one in reaction SMARTS is used. For mapping of the atoms in the reactants a circumflex is used instead of the colon in the reaction SMARTS. In the products, the mapping of the atoms is stored implicitly by their order starting with one in the transform pattern of the CHMTRN/PATRAN language.

The transpiler is written in Python. With the use of regular expressions, the LHASA transform pattern is split into its components. Each component is classified into the following types: ATOM, PROPERTY, BOND, FUSION, START_SIDECHAIN, END_SIDECHAIN, and RING.

The components are stored in the same order as in the LHASA transform pattern. As a result of the classification of the component by type, it is possible to create a graph representing the covalent structure reflected in the component list. The atoms, along with their properties, are stored within a node list, while bonds, combined with their associated properties, are stored in an edge list. The CHMTRN/PATRAN language uses the same syntax for indicating branches as the SMARTS language, utilizing parentheses. For easier translation, the branching level of an atom is stored in the node list. Zero indicates the main chain, while a positive number indicates the branching level. A similar syntax is used for indicating ring closures. The ring closures are stored as an edge in the edge list. Because molecular graphs are undirected, every edge is stored once. The orientation of edges serves to distinguish between sequential and ring closure bonds. Sequential bonds are represented by edges with start nodes preceding end nodes, whereas ring closure bonds are indicated by edges with start nodes succeeding end nodes. Based on the translation map, the atoms, their properties, and bonds are translated to the corresponding SMARTS expressions. In addition, the bond properties are transferred to the adjacent atoms.

Note that the elements in the LHASA transform pattern and SMARTS have the same order which remains unchanged during the translation process. Thus, the SMARTS/reaction SMARTS pattern can be generated by iterating the node list sequentially. Parentheses indicating the branches are set according to the level of the branch. Each step of the translation process is shown in Fig. 1. Due to special requirements of the topological fragment space creation process, the number of bonded hydrogens is the only allowed property besides the bond and atom types in the product pattern of the reaction SMARTS. Therefore, other properties and recursive expressions are not included. After the translation of the LHASA transform patterns to reaction SMARTS patterns, some of the patterns are slightly modified, because it is necessary to explicitly define the aromaticity of the atoms in the product pattern when a ring is formed.

#### Atom properties

While several atom properties can be translated literally to SMARTS, for some there is no direct counterpart. In these cases, recursive SMARTS expressions are crafted manually and stored in the translation map. The map finally contains the direct translation of functional groups to SMARTS expressions, but also synonyms or collections of functional groups. The mapping for atoms and atom properties can be found in Table S6–S9.

If multiple atom properties have to be combined in the SMARTS pattern, the logic is resolved as follows. In the context of atom properties, the keyword FGS (functional groups) encodes the verification of whether one of the following functional groups needs to be present. Thus the recursive SMARTS expressions have to be combined with the OR operator. When checking the absence of those groups, the AND operator is necessary to ensure that none of these functional groups is present. In LHASA transform rules, one can also check if the number of bonded hetero atoms or hydrogen atoms is more or less than a specific number, in addition to checking the exact number. While RDKit allows the definition of ranges of numbers for properties^25^, this is not possible in the original SMARTS language as defined by Daylight. To guarantee maximal compatibility, it is necessary to enumerate all possibilities in this range.

#### Bond properties

**Fig. 2:**
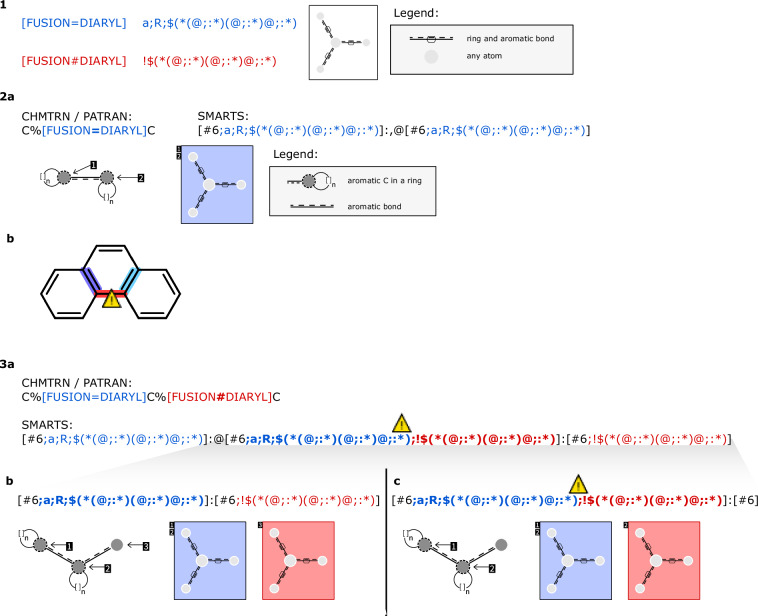
The translation of the fusion bond properties to SMARTS. (1) The translation of the CHMTRN/PATRAN bond properties DIARYL fusion bond and not a DIARYL fusion bond to SMARTS as atom properties. (2a) An example translation of the fusion bond property to the SMARTS pattern. (2b) The translation as SMARTS atom properties is not able to cover all cases. In this case the pattern has three matches on the example molecule. The bond highlighted in red is not a fusion bond, but the SMARTS pattern identifies it as fusion bond. (3a) An example translation of the negated fusion bond property into SMARTS patterns. This could lead to contradictory SMARTS patterns, if the negated fusion bond property is assigned to both incident atoms. In the example both the recursive and the negating recursive SMARTS expression would be assigned to the second atom and cause the contradiction. (3b) To cover all cases, the bond property must be assigned once to one and in a duplicated pattern to the other adjacent atom.

In SMARTS, bond properties are limited to bond type and cyclicality. Accordingly, it is not possible to translate the additional CHMTRN/PATRAN bond properties directly to SMARTS expressions. Where feasible, recursive SMARTS patterns were designed to transfer bond properties to adjacent atoms. Note that not all bond properties can be mapped this way. Fortunately, the fusion bond property, which is used to check for bonds shared by two or more rings in fused ring systems, is the only bond property used in the available LHASA transforms. The translation of the bond properties is available in Table S10–S12. There are some exceptions where the SMARTS pattern is not able to check reliably for fusion bonds, e.g. in poly-cyclic fused ring systems (see also Fig. 2a). Assigning the bond property “bond is not a fusion bond” to both incident atoms, certain edge cases remain uncovered. This method even produces SMARTS patterns that are inconsistent in certain cases. Most of these issues can be resolved by assigning the property to either one node or the other. To ensure comprehensive coverage, both assignment options are considered. Consequently, two sub-reactions are generated for each of these bonds. This special case is shown in Fig. 2b.

#### Adjustments to the transforms

There are transform patterns in which the product patterns contain more information than the reactant patterns. In the SAVI-Lib-2020 generation process, just as in SAVI-Space, the patterns are first applied as forward synthesis to the building blocks to obtain the products. However, an additional step has been taken to create SAVI-Lib-2020. The transform patterns are applied again, but to the products as retrosynthesis. All products are excluded if they either do not match the product pattern or result in reactants different from those originally used. Therefore, unlike with the SAVI space generation process, all information from the product patterns is also taken into account here. For this reason, it was necessary to manually transfer the information from the product pattern to the reactants wherever possible. This is particularly important for the Copper[I]-catalyzed azide-alkyne cycloaddition (Transform 2875), since depending on the catalyst used, either 1,4 (copper catalyst) or 1,5 (ruthenium catalyst) substituted triazoles are generated^26^. This is only considered in the product pattern, but not in the reactant pattern. Another case is the generation of non-favored valence states such as a carbon atom with a valence of 5 or a nitrogen atom with a valence of 4. However, this is already considered when creating the fragments of the fragments space. Reactants that will form products that have a non-favored valence state will not be considered. But this information can be crucial to obtain a single valid match, in the case the reactant pattern matches on more than one substructure of the reactant and is sorted out beforehand. Additional adjustments where made to the pattern, used to create the SAVI-Space-2020 and SAVI-Space-2024 in order to avoid unacceptable products, e.g. due to the ring tension.

### KILL statements

The KILL statements are used to filter unstable or sterically hindered products. For the SAVI-Lib-2020 the KILL statements were applied to the products, but in the fragment space creation process, it is not possible to apply changes to individual products. Therefore, the KILL statements were translated to filter rules, which are applied to the reactants.

#### Translation of the KILL statements

**Fig. 3:**
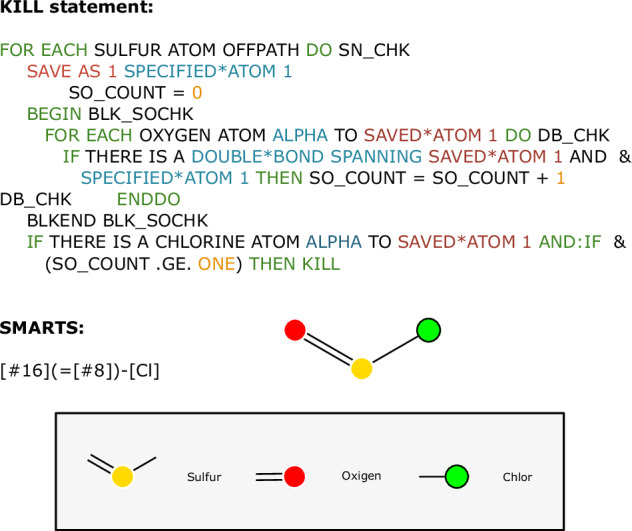
One complex KILL statement and the corresponding SMARTS pattern. This pattern was taken from the Mitsunobu Imide reaction (Transform 6043). The OFFPATH keyword indicates that the KILL pattern is only triggered if the participating atoms are not connected by bonds specified in the reaction smarts pattern.

The KILL statements are written in a natural language-inspired programming language and are applied to the products in sequential order. With the use of conditional statements (e.g. IF … ELSE) it is possible to check for specific structures either at certain positions (e.g. BETA TO ATOM*2) or anywhere (keyword: ANYWHERE) in the product. Additionally, it is possible to count specific cases or occurrences and even to iterate atoms in specific structures such as rings (e.g. THE RING CONTAINING ATOM*4). The combination of all these conditional statements enables nested and specific checks. But most of these KILL statements used in the LHASA transform rules concern explicit structures of a pattern present at a certain position or anywhere in (or not in) the product. Those statements are easily translatable to SMARTS expressions. Because there are no explicit rules for the order of the keywords in the KILL statements and the high number of conditional statements, it was decided to translate the approximately 300 KILL statements by hand. The translated KILL patterns can be found in the corresponding code. Furthermore, there are some complex statements with a bunch of conditional statements that could be translated to a short SMARTS pattern as can be seen in Fig. 3.

The complexity of the KILL statements is in no relation to the complexity of the corresponding SMARTS expressions. On the one hand, as in Fig. 3, the corresponding SMARTS pattern could be straightforward. On the other hand, there are keywords like “IN THE SAME RING AS …”, where, in the translation to SMARTS pattern every possible distance between atoms in the ring has to be checked. Apart from that, there are some keywords in the CHMTRN/PATRAN language that are not translatable to SMARTS expressions at all. One example is “LESS*HINDERED”, where the properties of two atoms, depending on the surroundings, have to be compared. As in the LHASA transform patterns, there are bond properties in the KILL statements, where the translation into SMARTS expressions does not cover all cases. For example, the WITHDRAWING*BOND keyword results in four different cases of electron-withdrawing groups at certain participating atoms that have to be checked. And even then, there are some cases which are not covered. Sometimes it is not possible to translate one KILL statement straight to one SMARTS expression, and it requires a list of SMARTS expressions. The issues faced for special bond properties are visualized in Fig. 2, and also for the keyword “IN COMMON RING” or “IN THE SAME RING AS …”, where various distances must be queried. In this case, we have opted for a maximum of six ring-bonds. Besides this, there is the possibility of GOTO statements in the KILL statements. This is used to jump to a specific block of the KILL statements. All these translated SMARTS expressions for each transform are held in a JSON file. For each of the translated SMARTS expressions, additional information is stored. For better readability, the SMARTS patterns for one transform are enumerated, especially for the guidance of the GOTO statements. In addition, whether it is an ONPATH or OFFPATH statement is stored, as well as the atoms participating in it. With this, it can be easily determined if one expression depends on both reactants. Furthermore, for GOTO instructions a SMARTS expression is marked with a “GOTO” key alongside a number indicating the index of the SMARTS expression to go to if triggered.

#### Application of the KILL statements

**Fig. 4:**
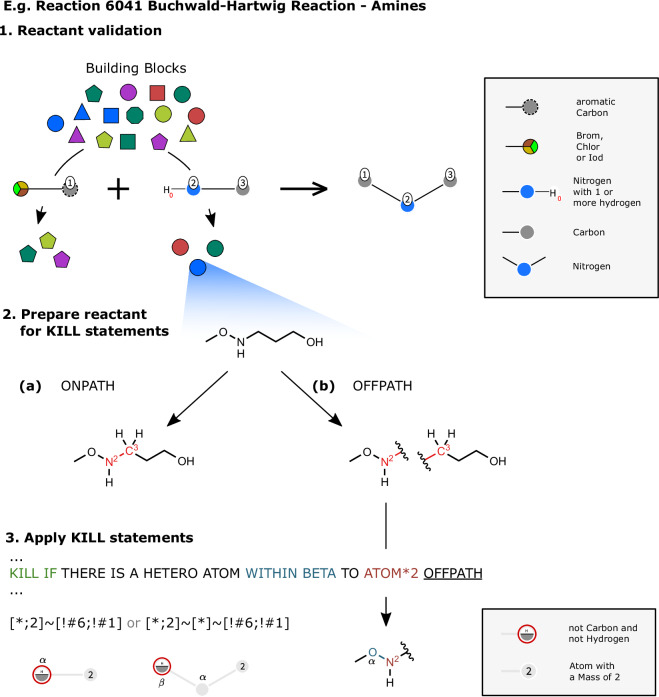
The filtering process of the reactants: 1. Suitable building blocks are selected based on the reaction pattern. 2. The preparation of the reactants for the application of the KILL statements. The reactant is prepared twice, once with (**a**) the masked leaving group and the enumerated backbone (red), and once with (**b**) an additional mask on the backbone. 3. The application of the KILL statements on the prepared reactants. In this example, the KILL statement is triggered by the presence of a hetero atom alpha to atom 2 (blue). So the building block is filtered out for this reactant in the Buchwald-Hartwig reaction of amines (Transform 6041).

Fragment spaces rely on the idea that all reactants associated with a reaction can be combinatorially combined without the exclusion of individual products. Not surprisingly, there are KILL statements that depend on both reactants, two in the Hantzsch thiazole synthesis (Transform 1171), three in the allene [2+2] cycloaddition (Transform 1391), and one in the Kabbe synthesis of 4-chromanones (Transform 2269). Thus it is necessary to divide the reactions into sub-reactions to cover as many combinations as possible. For example the KILL statement condition “IF CARBON ON ALPHA TO ATOM*4 OFFPATH AND:IF HYDROGEN ON ATOM*2 THEN” is triggered by the presence of a hydrogen atom on ATOM*2 and the absence of a carbon atom at the alpha position to ATOM*4 in the product. But in the reactants, ATOM*2 is part of the first reactant and ATOM*4 is part of the second reactant. Additionally, the condition triggers a GOTO statement, which leads to even more combination possibilities and thus to more subdivisions. To cover all cases without producing duplicates, a nested list holding all possible paths through the KILL statements is created for each reactant. Reactants with equal nested KILL lists are grouped, and paired with compatible groups of the other reactants. When the KILL statements are applied to the reactants as SMARTS expressions, the parts of the reactant that are not present in the product must be masked. Each KILL statement is either ONPATH or OFFPATH. ONPATH statements can be applied directly to the reactant with the leaving group masked. With OFFPATH statements, on the other hand, the reactions SMARTS pattern must also be taken into account. OFFPATH statements must not be applied to contiguous subgraph parts of the reactant that match the reaction SMARTS pattern. Therefore, each reactant must be represented in two different ways: first, as a variant with the masked leaving group and the numbered substructure corresponding to the atom mapping of the reaction SMARTS pattern; and second, as a variant with additional transformations in the substructure. The simplest way to generate the variant for OFFPATH statements is to cut all bonds that connect atoms corresponding to the atom mappings of the reaction SMARTS pattern. For the enumeration of the atoms according to the atom mapping in the reaction SMARTS, the atomic mass property of the SMARTS and SMILES language is used. The exact process is shown in Fig. 4.

### Aromaticity

The difference between the aromaticity models in the CACTVS toolkit^27^ on the one hand and in the RDKit toolkit^28^ and NAOMI library^29^ on the other hand emerged as the most important issue while translating and applying the LHASA transform rules. In the CACTVS toolkit, ring systems with exocyclic double bonds are not considered aromatic, and with this, possible tautomeric structures, where the pi-Electron of the exocyclic double bond is shifted in the ring are ignored. In the NAOMI and the RDKit toolkit, these ring systems are still considered aromatic when the atom bonded through the exocyclic double bond is oxygen, nitrogen, or sulfur. This different view of aromaticity leads to different outcomes of the reactions when applying the LHASA transform rules. The goal for the SAVI-Space-2020(Lib-2020 rules) is to create a chemical space that is as similar as possible to the original SAVI Library. We therefore added the option to use the CACTVS aromaticity model to the NAOMI code base for the creation of SAVI-Space-2020(Lib-2020 rules). The aromaticity of ring systems is still a topic of discussion^30,31^, especially because aromaticity is not directly measurable^32^. Therefore, it was decided to give the user the option to choose between the CACTVS aromaticity model and the NAOMI aromaticity model resulting in two variants of SAVI-Space.

### Building blocks

The building blocks were standardized by removing explicit hydrogens, disconnected metals, and salts. Furthermore, sulfoxides were converted to their uncharged form and carboxylates are protonated. Like in the SAVI-Lib-2020, all organometallic compounds were removed. In addition, all building blocks with a molecular weight of more than 700 were discarded to avoid oversized products. For each of the 53 transforms used to define the SAVI-Lib-2020 — a list of the transforms and their names can be found in Table S1 — the number of matches for each reactant of the translated transform rule was counted. Only building blocks matching exactly one of the two reagent patterns, and this one also only once, were selected. Components that match both reaction patterns can lead to product mixtures or even polymerization in real-world conditions. This procedure was adopted from SAVI. In SAVI-Space, there is additional handling such that when reactants match both reactant patterns but one match is excluded by a KILL statement, the other match will still be considered in the SAVI-Space creation process. There are exceptions in SAVI-2020 regarding duplicate handling. For example, there are symmetrical patterns, such as the alkene pattern in the alkene [2+2] cycloaddition (Transform 1391) and Suzuki-Miyaura Cross-Coupling of alkenes (Transform 6009), which double-match all alkenes. Nevertheless, the SAVI-Lib-2020, as well as the SAVI-Space-2020(Lib-2020 rules) consider the building blocks with alkenes for the transform rules by unifying the matches. In the publication by Patel *et al*. is stated: “Counts were adjusted for duplication in products due to alkene reactivity at both ends of the bond (ID 6009)”^15^. In the SAVI-Space-2020 and SAVI-Space-2024 creation process, matches are not unified. Each reaction is first analyzed for its behavior producing possible product mixtures. In some SAVI transforms, different matches may belong to the same reaction center and, as a result, lead to the same product instead of product mixtures. To ensure unique matches, a subset of nodes is selected from the reaction pattern based on their distinct roles in the reaction. This helps to avoid situations where multiple mappings could lead to the same product, ensuring that only one unique transformation is considered for each reaction. Future versions could integrate additional chemical knowledge to refine the transform rules accordingly.

### Reaction-driven fragment space creation

The variants of the SAVI-Space are encoded as topological fragment spaces^8^ as well as the traditional fragment space created with the CoLibri toolkit^33^. Different sets of reactants form nodes and the connection rules built from the reaction patterns form edges in so-called topology graphs. One topology graph is defined for each reaction pattern. Algorithms for the fingerprint similarity search^8^, a comparison of spaces^34^, and the calculation of property distributions^35^ are all based on the topological fragment space. The substructure search^9^ is based on the traditional fragment space.

### Protecting groups

In chemical reactions, protection groups are necessary to prevent functional groups from reacting in an undesired manner. Some of the Enamine Building Blocks come with protecting groups. At the last step of the SAVI-Lib-2020 product generation, the products were checked for the presence of protecting groups. If a product has one or more protecting groups, the complete unprotected version of this product is added to the collection. The following structures were used for the protection of amin and hydroxyl groups. Amino protecting groups: tert-Butoxy carbamate (Boc), fluorenylmethyloxycarbonyl (Fmoc), benzyloxy carbamate (CBz). Carboxyl protecting groups: tert-Butyl ester (t-Bu ester), benzyl ester (Bz ester). Hydroxyl protecting groups: tert-Butyl ether (t-Bu ether), benzoate (Bz)^15^. For the SAVI-Space, the fragments must be checked for the presence of protection groups during the fragment space creation process, prior to assembling products. For each fragment deemed applicable to a specific reaction, if a protection group is present, the fragment is stored with the protection group removed.

### Predicted properties

In the SAVI-Lib-2020, each product was annotated with more than 60 properties. Besides the data about the building blocks and the reaction used, other properties of the products are calculated during library creation. Some of these properties are given by the SCORE statements in the LHASA transform rules, and some of the properties were calculated by the CACTVS toolkit. In the SAVI-Spaces the SCORE statements are not yet used, so there are no such properties included. For the analysis of the entire Space, SpaceProp is able to calculate property histograms without explicit compound enumeration. Distributions of properties like molecular weight, clogP, or the number of rotatable bonds in a fragment space^35,36^ can be determined. The resulting property histograms for SAVI-Space are shown in Fig. 10 and Figure S1.

### Hardware and runtime

The creation of the SAVI-Spaces was done on a standard desktop PC (i.e. Intel(R) CORE(TM) i5-8500 CPU @ 3.00 GHz with 6 cores). The filtering process of the building blocks for the final SAVI-Space-2024 took about 7.5 hours. Based on these pre-processed molecules, the creation of the topological fragment space took an additional 1.4 hours and the creation of the traditional fragment space took 1.1 hours. This results in a total runtime of 10 hours for the creation of SAVI-Space-2024. The creation of the SAVI-Lib-2020, by comparison, required massive computational resources and time. The creation of SAVI-Lib-2020 was performed on an HPC cluster with several thousand cores and took several millions of CPU hours^15^. Parts of the computing time went into the calculation of properties. As described above, SpaceProp^35,36^ may be used to also calculate property distributions, which takes only a few minutes for the entire SAVI-Space.

## Data Records

The dataset is available at the research data repository of the University of Hamburg^37^. The SAVI-Space-2020(SAVI-Lib-Rules), SAVI-Space-2020 and SAVI-Space-2024 are available as space files (SAVI-Space[...].space) and can be opened with SpaceLight for fingerprint similarity search^38^ and SpaceMACS for substructure search^9^, as well as SpaceProp2^36^. Additional the preprocessed building blocks are available for the SAVI-Space-2020(SAVI-Lib-Rules) and SAVI-Space-2020.

### Building blocks

For SAVI-Space-2024, the original dataset comprised 288,748 Enamine Building Blocks (state July 2024), from which 255,861 compounds were retained after filtering. We created SAVI-Space-2020(Lib-2020 rules) based on the original 155,129 Enamine Building Blocks from December 2019 used for the creation of SAVI-Lib-2020. After filtering, based on the SAVI-Lib-2020 rules 138,966 building blocks were used to create this version of the space. For the creation of SAVI-Space-2020 137,982 building blocks were used. Transforms containing multiple patterns were split into separate reactions for the generation of SAVI-Space. Additionally, there are those transforms where one original LHASA transform pattern leads to multiple translated reaction SMARTS patterns because of issues addressed in Section “Translating the LHASA transform pattern”. In summary, the whole procedure results in 109 sub-reactions in SAVI-Space-2024. Based on these, there are 218 collections of processed building blocks, one for each of the two reactants in each sub-reaction.

### Generated products

The final SAVI-Space-2024 dataset comprises over 7.5 billion molecules, organized into 86 distinct subsets, each associated with one of the 53 transforms of SAVI. Since the number of building blocks increased from 155 thousand to 278 thousand between 2019 and 2024, the number of products increased substantially. The resulting database is 0.8 GB in size. The SAVI-Space-2020(Lib-2020 rules) contains more than 2.3 billion molecules. It is approximately 33% larger than the size of the SAVI-Lib-2020. An overview of the created SAVI-Spaces is shown in Table 2. A more detailed comparison of the SAVI-Lib-2020 and the SAVI-Space-2020(Lib-2020 rules) follows below.

**Table 2:** Size of the SAVI-Spaces based on the Enamine Building Blocks from December 2019 and July 2024 and on the different handling of patterns and rules with respect to aromaticity.

Version	building blocks used	number of products	number of products without KILL	computational time*	storage size
SAVI-Space-2020 (Lib-2020 rules)	1.39 × 10^5^	2.34 × 10^9^	3.65 × 10^9^	3 h	2.1 GB
SAVI-Space-2020	1.38 × 10^5^	2.40 × 10^9^	3.34 × 10^9^	3 h	0.8 GB
SAVI-Space-2024	2.56 × 10^5^	7.55 × 10^9^	1.07 × 10^10^	10 h	1.4 GB

* Intel(R) CORE(TM) i5-8500 CPU @ 3.00 GHz.

## Technical Validation

### Overlap between SAVI-Lib-2020 and SAVI-Space-2020(Lib-2020 rules)

**Fig. 5:**
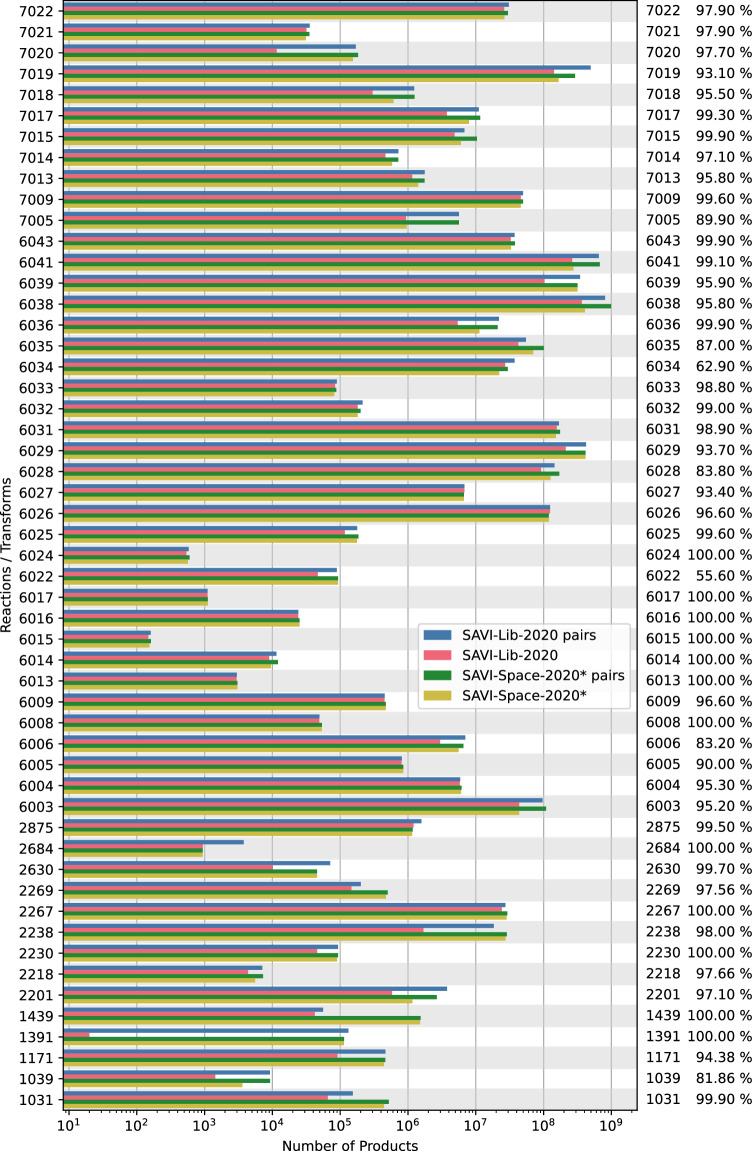
Comparison of the number of the Products, per transform rule, in the SAVI-Lib-2020 and the SAVI-Space-2020(Lib-2020 rules). For both implementations the number of products before (pairs of reactant combinations) and after applying the KILL statements is shown. On the right hand side of the figure, the percentages of test sets of a maximum of 1000 randomly selected products per transform are shown.

Both, the library (SAVI-Lib-2020) and the space (SAVI-Space-2020 Lib-2020 rules) are based on the same Enamine Building Blocks from December 2019. There are several reasons why compounds might be contained in the library but not in the space or vice versa, such as differences in structure interpretation and pattern matching between the toolkits employed, the applicability of KILL statements on the reactant versus product level, or differences in the specificity of the CHMTRN/PATRAN transforms and the corresponding Reaction SMARTS. To get an understanding of the influence of the KILL statements, the comparison was performed on SAVI-Space before and after applying them. The results are shown in Fig. 5 and Table S2. By and large, the number of products in the SAVI-Lib-2020 and the SAVI-Space-2020(Lib-2020 rules) are on the same order. To estimate the coverage of SAVI in SAVI-Space, test sets of a maximum of 1000 products per reaction were randomly generated. By using the SpaceLight fingerprint similarity search algorithm^8^ and the connected subgraph fingerprint (CSFP)^38^, the number of possible matches of the products in the SAVI-Space could be defined. The exact match is verified by comparing the canonical SMILES strings of the query molecule and the match in the SAVI-Space. The evaluation of how many SAVI-Space-2020(Lib-2020 rules) products can be found in the SAVI-Lib-2020 could not be easily done because of the enormous size of the library, which can only be searched sequentially. Therefore, only the number of products in both implementations was compared. The results are shown in Fig. 5. In addition to the data presented in Fig. 5, the corresponding values can be found in Table S2. With a few exceptions, for most transforms, a coverage of above 95% is achieved, for roughly half of the transforms the coverage is over 99%.

Figure 5 shows that one of our goals was achieved, namely that SAVI-Space-2020(Lib-2020 rules) is a good mimic of the SAVI-Lib-2020. Comparing the number of reactant pairs (potentially leading to product molecules) in the SAVI-Lib-2020 and the SAVI-Space-2020(Lib-2020 rules) reveals very similar values. This means that the reaction patterns were successfully translated as reaction SMARTS. Not surprisingly, the difference in the number of valid products is slightly higher after applying the KILL statements. As outlined before, these apply to individual product molecules for the SAVI-Lib-2020 and can be approximated to some degree only, using reactant filtering for the SAVI-Space-2020(Lib-2020 rules). Generally, SAVI-Space-2020(Lib-2020 rules) contains a higher number of products than the SAVI-Lib-2020. The difference varies significantly from reaction to reaction but in total SAVI-Space-2020(Lib-2020 rules) contains 34% more product molecules than the sibling Library. By extrapolating from the sample number of products of the Library found also in the Space, we may estimate a 95% coverage. The other way round, approximately 71% of SAVI-Space-2020(Lib-2020 rules) products are contained also in the Library.

#### Limitations of KILL statements and SMARTS translations

Despite the high degree of overlap of product molecules from both implementations, there are some differences between these compound collections. The biggest differences are because the KILL statements cannot be applied successfully for the chemical fragment space (SAVI-Space-2020 Lib-2020 rules) in all cases. For example, the keyword “ENOLIZABLE” in the Wittig via methoxy-ylide reaction (Transform 7020) could not be fully covered by the SMARTS expressions. The keyword “LESS*HINDERED” in the allene [2+2] cycloaddition (Transform 1391), arylpyridines synthesis via o-aminocarbonyl (Transform 2201), and the Pictet-Spengler reaction (Transform 2238) could not be translated to SMARTS expressions at all.

#### Variations in aromaticity models

Besides that, some differences are caused by the different handling of aromaticity in the CACTVS toolkit — that was used for the creation of the library —, the RDKit toolkit, and the NAOMI library — used for the chemical space creation — as well as handling of aromaticity when it comes to functional groups. Although the aromaticity model of the CACTVS toolkit was adopted to create the chemical fragment space, there are still differences in handling the bond types in aromatic ring systems. By our adopted aromaticity implementation, the bonds in these ring systems are considered as either single or double bonds implicitly ensuring correct chemical structures by atomic valence states.

### Different interpretations of LHASA rules: Lib-Rules vs Space-Rules

The different handling of duplicates is one key difference when it comes to the creation of the final Spaces compared to the Library rules. For example in the Chan-Lam coupling (Transform 7022), the CHMTRN/PATRAN pattern allows primary and secondary amines, but not tertiary amines. Due to the composition of the pattern, it always matches two times on the secondary amines so that only reactants with primary amines are considered for creating the products. In SAVI-Space-2020(Lib-2020 rules) this behavior was imitated, but in the SAVI-Space-2020 and SAVI-Space-2024, this behavior was excluded by the different handling of duplicates, so secondary amines are also taken into account. Moreover, in the Suzuki-Miyaura Cross-Coupling reaction of iodo (Transform 6005), bromo (Transform 6006), and alkenes (Transform 6009), and the sulfonamide formation under Schotten-Baumann conditions from aryl bromide (Transform 6029), for some of the SAVI products, the pattern for the first reactant matches both reactants. This is excluded during the creation of SAVI-Space because this reaction can lead to product mixtures or even polymerization in real-world conditions. In the Hantzsch thiazole synthesis (Transform 1171), and the Liebeskind-Srogl thioamide coupling (Transform 6022), there are products in the SAVI-Lib-2020 that contain an uncharged nitrogen atom with a valence of 4. These products are not found in any SAVI-Spaces because this valence state is not allowed in the NAOMI valence state model. Additionally, it seems to be a different handling of halogens or alcohols bonded to an aromatic ring system or a double bond. Products that should usually face KILL statements including halogens, for example, the keyword GOOD*LEAVING, are still present in SAVI-Lib-2020 when they are bonded to an aromatic ring system or to a carbon that is double bonded. By analyzing the products of the SAVI-Space-2020(Lib-2020 rules), there are cases of products found that are unlikely to exist. The most frequent case is a possible creation of ring tension e.g. in the Paal-Knorr pyrrole synthesis (Transform 1031), Feist synthesis of pyrroles (Transform 1039), pyrazoles sythesis from beta carbonyl carboxylic acid derivatives (Transform 1439), Pictet-Spengler reaction (Transform 2238) and the Kabbe synthesis of 4-chromanones (Transform 2269). These products are also present in SAVI-Lib-2020. The authors of the SAVI-Lib-2020 are already aware of these issues and have updated the LHASA transform rules to prevent the creation of these products in a future version. In the new rules for SAVI-Space-2020 and SAVI-Space-2024 the pattern has been changed to prevent these products.

### Impact of these interpretations on rule handling

**Fig. 6:**
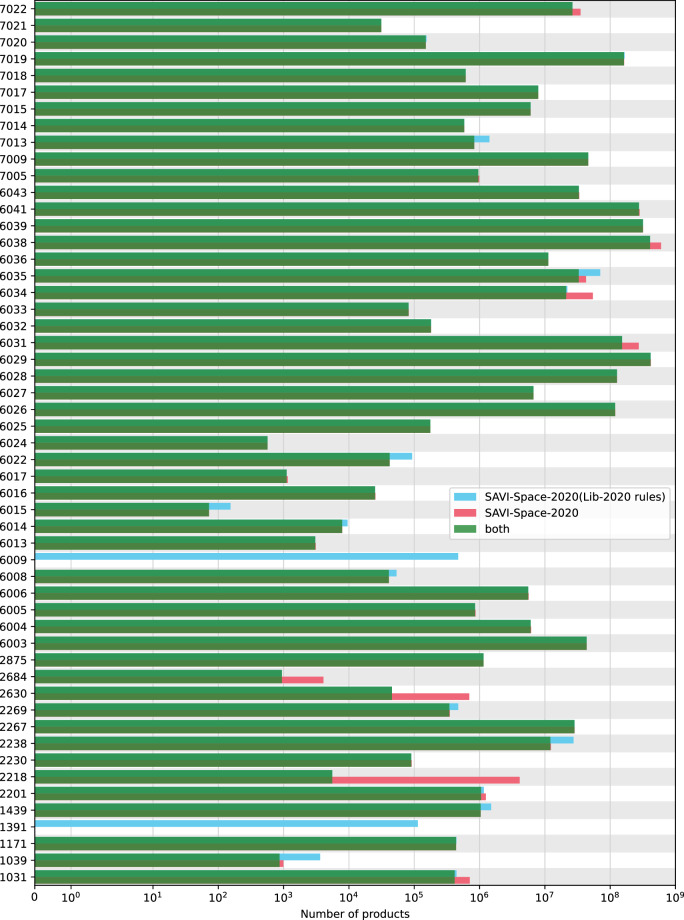
Comparison of the number of products and the overlap in SAVI-Space-2020(Lib-2020 rules) and SAVI-Space-2020. The products that are present in both SAVI-Space-2020(Lib-2020 rules) and SAVI-Space-2020 are shown in green and visually obscure the blue bars, indicating the number of products in SAVI-Space-2020(Lib-2020 rules), and the number of products in SAVI-Space-2020 in red.

**Fig. 7:**
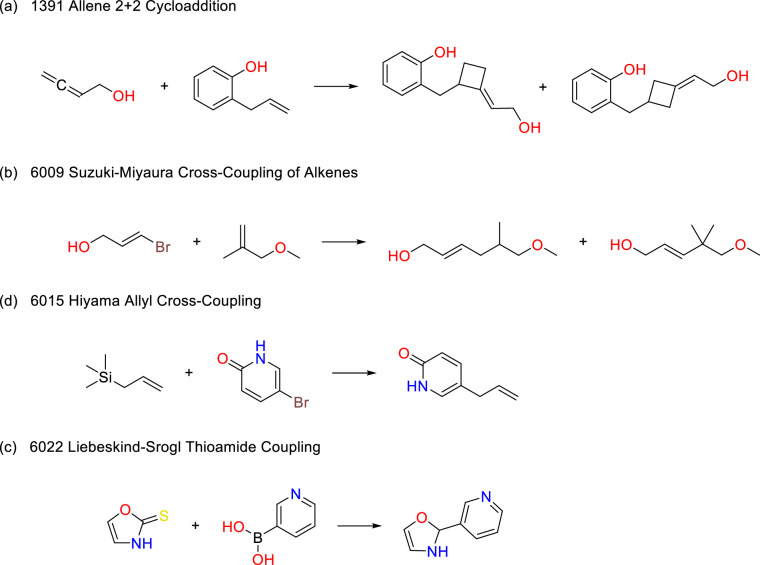
Products that are only present in the SAVI-Space-2020(Lib-2020 rules). (**a**)-(**b**) No product was generated for the SAVI-Space-2020 at all, since the symmetrical patterns of the reactants from the Lhasa transform rules always match twice and thus could generate product mixtures. The ring systems in the second reactant in (**c**), and in the first reactant in (**d**), are not treated as aromatic in the SAVI-2020 rules because of the exocyclic double bond to oxygen and sulfur.

**Fig. 8:**
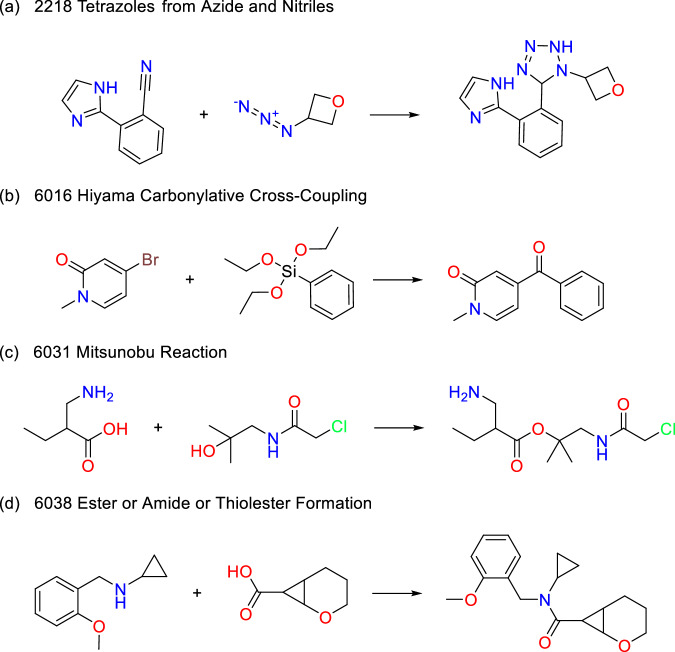
Products that are only present in the SAVI-Space-2020. (**a**) In the product pattern of the LHASA transform pattern, it is ensured that the generated tetrazole ring is only substituted once. This was not transferred to the reactant pattern. Since only the properties of the reactants are used for the creation of the SAVI spaces, the SAVI-Space-2020(Lib-2020 rules) also contains molecules with double-substituted tetrazole rings. In the SAVI-Space-2020, this was adjusted to the reactant pattern. Therefore, only single substituted ones are present here. (**b**) The aromatic ring system in the first reactant is defined as non-aromatic in SAVI-Space-2020(Lib-2020 rules) because it contains an exocyclic double bond to an oxygen. (**c**) The SMARTS pattern of the second reactant (simplified as C-C-[O;H1]) generates double or triple matches when the alcohol group is attached to a tertiary or quaternary carbon atom, an issue that has been resolved in the SAVI-Space-2020 and SAVI-Space-2024. (**d**) The SMARTS pattern of the first reactant (SMARTS simplified: C-[O,S,N;H0]) produces double matches because oxygen, nitrogen, and sulfur atoms without hydrogen tend to form multiple bonds to carbon atoms, resulting in multiple valid matches in the substructure. This problem was overcome in the SAVI-Space-2020 and SAVI-Space-2024.

As shown in Table 2, the SAVI-Space-2020(Lib-2020 rules) contains about 12% fewer products than the SAVI-Space-2020 following our adjusted rules. A direct comparison for each transform is shown in Fig. 6. Here, each transform and thus single-space is compared and the overlap is shown visually. For the allene [2+2] cycloaddition (Transform 1391) and the Suzuki-Miyaura Cross-Coupling reaction of alkenes (Transform 6009), SAVI-Space-2020 leads to no products. This is caused by the symmetrical alkene pattern for one reactant. A product mixture will be obtained because of the double match at the symmetrical pattern. Any exclusion of one of the products due to possibly triggered KILL statements is not taken into account. It is easy to see that for most transforms there is still a good coverage of SAVI-Lib-2020 if we use our rules, but additional products are generated. One reason for this is that some valid matches that do not generate product mixtures still match two or more times due to the structure of the reactant pattern, such as the secondary amine in the Chan-Lam coupling (Transform 7022). Some examples of products that are only available in SAVI Space-2020(Lib-2020 rules) are shown in Fig. 7. Similarly, products that are only available in SAVI-Space-2020 are shown in Fig. 8.

### Overlap with other chemical spaces

While it is close to impossible to compare two billion-sized libraries to determine their overlap - even if it is small, the method SpaceCompare^34^ is able to list all identical product molecules from fragment spaces as long as the overlap is not too big. In a first step, the SpaceCompare algorithm compares only the fragments of two spaces. Fragments that are unique to one of the spaces are sorted out, thus considerably reducing the number of products that actually need to be compared in the second step. Since the Enamine REAL Space^39^, the eMolecules eXplore^40^, and the the Freedom Space^41^ contain too many identical fragments as does the SAVI-Space-2024, SpaceCompare stopped after step 1 and therefore no results are available here. Note that due to differences in reaction patterns, identical fragments may well lead to different products, so the actual overlap remains unknown for these spaces. However, Lessel *et al*.^42^ predicted the overlap of much older versions of these spaces as surprisingly small. The SAVI-Space was compared with the CHEMriya^43^, GalaXi^44^, and the Knowledge Space^45^. The comparison of the SAVI-Space-2024 with the chemical spaces is shown in Table 3. It turned out that the overlap is very small. For instance only 0.352% of compounds in SAVI Space are also in CHEMriya. The result is comparable for GalaXi and KnowledgeSpace.

**Table 3:** Overlap of the SAVI-Space-2024 with other fragment spaces, such as CHEMriya^43^, GalaXi^44^, and the Knowledge Space^45^.

Chemical Space	Products	Overlap
CHEMriya^43^	2.15 × 10^10^	2.64 × 10^5^
GalaXi^44^	1.24 × 10^10^	2.87 × 10^5^
Knowlegde Space^45^	2.92 × 10^14^	1.51 × 10^6^

### Synthetic accessibility

**Fig. 9:**
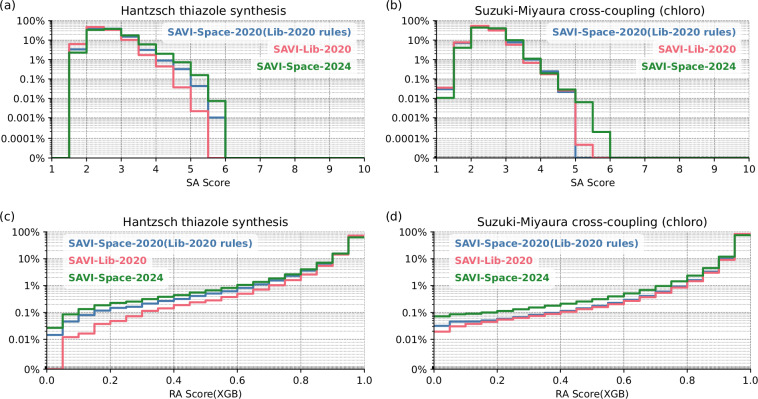
Distribution of the synthetic accessibility scores (SA-Scores) for the products of the Hantzsch thiazole synthesis. (**a**), and the Suzuki-Miyaura cross coupling (**b**), and retrosynthetic accessibility scores (RA-Scores) for the products of the Hantzsch thiazole synthesis (**c**), and the Suzuki-Miyaura cross coupling (**d**) of the different chemical spaces. The XGBoost (eXtreme Gradient Boosting) model was used to calculate the RA-Scores.

Since the computation of the LHASA score was not integrated in our implementation of the SAVI space, other assessments of synthesizability were used. In order to still have a reference point, the synthetic accessibility score (SA-Score)^46^ and the retrosynthetic accessibility score (RA-Score)^47^ were used for evaluation. Due to the enormous number of products, these scores were not calculated for the entire chemical space, but instead were applied to the Hantzsch thiazole synthesis (ring closure reaction) and the Suzuki-Miyaura cross-coupling (chloro) (open-chain reaction) as examples. The results are shown in Fig. 9. for the products of the SAVI-Lib-2020 as well as for the SAVI-Space-2020(Lib-2020 rules) and for the SAVI-Space-2024 as a histogram. Since the two scores used were created for the evaluation of different reactions, it cannot be ruled out that the scores obtained can be attributed to a different reaction type than that used in the example reaction. Since the selected reactions are well established in chemical synthesis, we assume that this influences the overall result only marginally.

The SA Score ranges from 1 (easy to synthesize) to 10 (difficult to synthesize). Ertl *et al*.^46^ roughly classified compounds with scores >6 to be “difficult to synthesize”. All molecules from the three variants of SAVI (see Fig. 9) have scores below this threshold. Thus, they are estimated to be comparatively easy to synthesize. Also the distribution of the scores for the SAVI-Lib-2020 and the two SAVI Space variants are very similar. Furthermore Ertl *et al*.^46^ observed a similar synthetic accessibility distribution for catalog molecules. Admittedly, the distribution shifts visibly towards worse scores for the SAVI-Space-2024. However, since the Y-axix is on a logarithmic scale, this provides a missleading impression, since only a small fraction of the molecules is affected.

The RA Score ranges between 0 (difficult to synthesize) and 1 (easy to synthesize). A score higher than 0.9 was predicted for 80% (Hantzsch thiazole synthesis) and 91% (Suzuki-Miyaura coupling) of the molecules in SAVI Space. For the SAVI-Lib these values are only slightly higher, an additional 4% of molecules for the Hantzsch thiazole synthesis and an addirional 2% for the Suzuki-Miyaura coupling.

Beside the computational assessment, we were interested to see if SAVI Space 2024 contains molecules that really have been synthesized and whether the synthetic route is the same as reported in literature. To achieve this, we used a recently published benchmark set of diverse bioactive ChEMBL compounds (Set S; Neumann & Klein)^48^. SAVI Space 2024 was screened with Tanimoto Fingerprint (fCSFP4) similarity for molecules identical with one of the benchmark ChEMBL compounds. 147 out of the 2917 compounds can be found in the SAVI-Space-2024. The number of known bioactive compounds in the space is quite high if one takes the typical estimates for the number of unique drug-like compounds into account. Since the general aim of combinatorial libraries is to span a chemical space of new chemical matter, SAVI-Space is certainly a useful collection for drug discovery purposes. By design, the focus is set on exploring new intellectual property rather than covering already existing compounds. The synthetic success of compounds encoded in SAVI Space is due to the use of robust chemistry. Discrepancies between the reaction pathways encoded in SAVI Space 2024 and the ChEMBL compounds do not necessarily indicate synthetic inaccessibility. The chosen synthesis route always depends on the accessibility of building blocks. With the update of the Enamines Building Block set, other, maybe easier synthetic routes might be possible. As aforementioned our assessment is just a spot check of a tiny subselection of SAVI Space 2024.

### Distribution of properties relevant for drug discovery

**Fig. 10:**
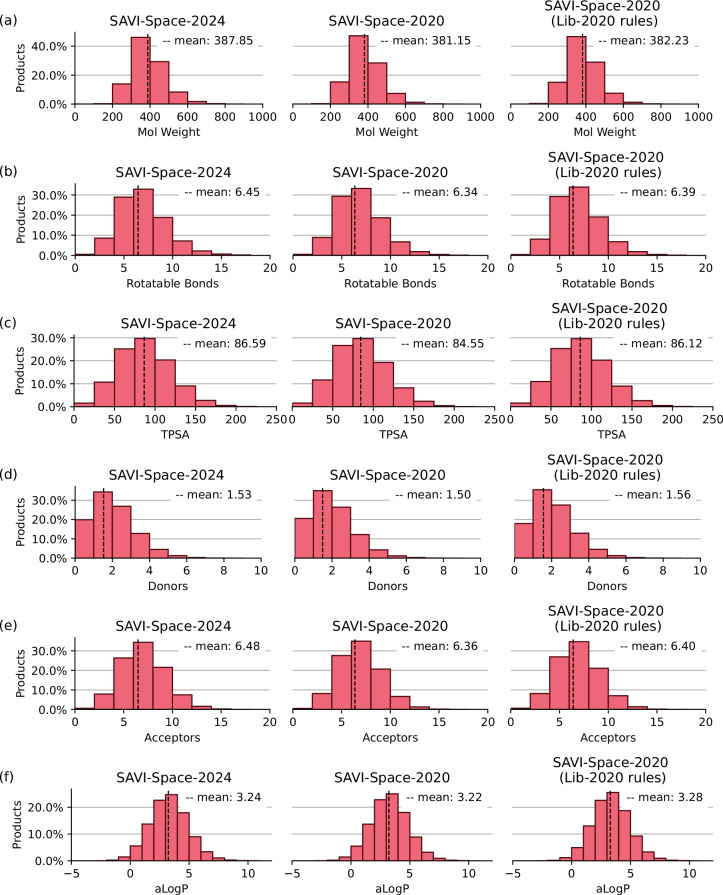
Distribution of properties of the different SAVI-Spaces. (**a**) Distribution of the molecular weight; (**b**) distribution of the number of rotatable bonds; (**c**) distribution of the topological polar surface area; (**d**) distribution of the number of hydrogen bond donors; (**e**) distribution of the number of hydrogen bond acceptors; (**f**) distribution of the aLogP.

The SAVI-Lib-2020 was generated with the aim to provide synthetic accessible molecules suitable for drug discovery. Patel *et al*.^15^ analyzed several physicochemical parameters important for oral bioavailability as described for instance by Lipinski *et al*. (molecular weight ≤500g/mol, number of hydrogen bond donors ≤5 or acceptors ≤10, logP ≤5)^49^ or Veber *et al*. (rotatable bonds ≤10, TPSA ≤140A)^50^. They confirmed that more than 80% of SAVI compounds fall within this range. As explained above, the different treatment of the LHASA rules has led to partially different number of products, and in addition the new version of building blocks has led to a much larger space. In order to clarify whether SAVI-Space-2024 is still a valuable source for drug discovery, SpaceProp2^36^ was used to calculate distributions of relevant properties (Fig. 10 and Figure S1). The mean for all calculated properties is shifted slightly towards higher values but the overall distributions remain similar. This makes SAVI-Space-2024 a valuable source for potential bioactive compounds as required for early-stage drug discovery.

## Usage Notes

The three variants of SAVI-Space are available at the research data repository of the University of Hamburg^37^. The reaction SMARTS as well as all KILL statements converted into SMARTS can be found in the respective directories. We provide all scripts to translate the LHASA transform patterns, filter the building blocks and recreate SAVI-Space for future adaption and reuse, for example on alternative building block collections at GitHub (https://github.com/rareylab/SAVI-Space). It is possible to only create the subsets of the building blocks for each reactant of each transform, based on Python and the RDKit. Note that tools from the NAOMI ChemBio Suite (UHH) or Colibri/InfiniSee (BioSolveIT GmbH) are required to run the final fragment creation and search in this fragment space. NAOMI tools are free for academic use and available from their official website (https://uhh.de/naomi/). Besides the tool for creating the fragment space, SpaceProp2^36^ can be used to analyse the fragment spaces, SpaceLight^8^ for fingerprint similarity search, and SpaceMACS^9^ for substructure search. The collection of building blocks used for the SAVI-2020 is available on the SAVI project webpage (https://cactus.nci.nih.gov/download/savi_download/savi_diversity/Dec2019_instock_BBs_155k_sdf.zip)^16^. Note that, due to legal constraints by external data providers, SAVI Space 2024 is in a data format that cannot be read with standard text editors.

## Supplementary information

SUPPLEMENTARY INFORMATION

## Data Availability

The code for the SAVI-Space creation process is available at (https://github.com/rareylab/SAVI-Space). The academic version of the NAOMI toolkit is available at (https://uhh.de/naomi/).
