# Correction: SAVI Space—combinatorial encoding of the billion-size synthetically accessible virtual inventory

**DOI:** 10.1038/s41597-025-05700-7

**Published:** 2025-07-31

**Authors:** Malte Korn, Philip Judson, Raphael Klein, Christian Lemmen, Marc C. Nicklaus, Matthias Rarey

**Affiliations:** 1 University of Hamburg, ZBH – Center for Bioinformatics, 22761 Hamburg, Germany; 2 Heather Lea, Bland Hill, Norwood, Harrogate, HG3 1TE England; 3 BioSolveIT GmbH, St. Augustin, Sankt Augustin, Germany; 4 NCI, NIH, CADD Group, NCI-Frederick, Frederick, Maryland, 21702 USA

**Keywords:** Virtual screening, Data processing

Correction to: *Scientific Data* 10.1038/s41597-025-05384-z, published online 23 June 2025

In this article the captions to Figures 6, 7, and 8 were inadvertently swapped.

In addition, the references to Fig. 7, Fig. 8, and Fig. 6 in the paragraph starting ‘Impact of these interpretations on rule handling’ should instead have referred to Fig. 6, Fig. 7, and Fig. 8, respectively.

The original article has been corrected.

