# Supplementary material for: SAVI Space—combinatorial encoding of the billion-size synthetically accessible virtual inventory
Source: Sci Data. 2025 Jun 23;12:1064. doi: 10.1038/s41597-025-05384-z (PMC12185686; doi:10.1038/s41597-025-05384-z)
Supplement: Supplementary file 1 — SUPPLEMENTARY INFORMATION [file 41597_2025_5384_MOESM1_ESM.pdf]

Supplementary Information

Contents

Supplementary Figures **2**
Figure S1.Distribution of the number of heavy atoms across different SAVI-Spaces. 2

Supplementary Tables **3**
Table S1. Names of the transforms. 4
Table S2. Number of products for the SAVI-Lib-2020 and SAVI-Space-2020(Lib-2020 rules). . . 5
Table S3. Number of products for each transform of SAVI-Space-2024. 6
Table S4. Number of products for each transform of SAVI-Space-2020. 7
Table S5. Number of products for each transform of SAVI-Space-2020(Lib-2020 rules). 8
Table S6. SMARTS translations of CHMTRN/PATRAN for atom. 9
Table S7. SMARTS translations of CHMTRN/PATRAN for functional groups. (Part 1) 10
Table S8. SMARTS translations of CHMTRN/PATRAN for functional groups. (Part 2) 10
Table S9. SMARTS translations of CHMTRN/PATRAN for synonym. 12
Table S10. SMARTS translations of CHMTRN/PATRAN for fusion. 12
Table S11. SMARTS translations of CHMTRN/PATRAN for no fusion. 12
Table S12. SMARTS translations of CHMTRN/PATRAN for bond. 13
Table S13. SMARTS translations of CHMTRN/PATRAN for charge. 13
Table S14. SMARTS translations of CHMTRN/PATRAN for positional statements. 13

Supplementary Figures

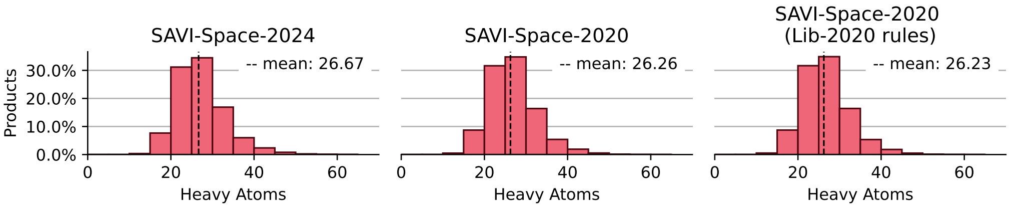

Figure S1: Distribution of the number of heavy atoms across different SAVI-Spaces.

Supplementary Tables

ID	Name
1031	Paal-Knorr Pyrrole Synthesis
1039	Feist Synthesis of Pyrroles
1171	Hantzsch Thiazole Synthesis
1391	Allene 2+2 Cycloaddition
1439	Pyrazoles from Beta Carbonyl Carboxylic Acid Derivatives
2201	Arylpyridines via o-Aminocarbonyls
2218	Tetrazoles from Azide and Nitriles
2230	Phthalazin-1-ones from 2-Acylbenzoic Acids
2238	Fused Aryl(2,3-HR)Pyridines (Pictet-Spengler)
2267	Sonogashira Coupling
2269	Kabbe Synthesis of 4-Chromanones
2630	Benzazepin-2-ones by Pictet-Spengler Reaction
2684	Benzo[b]furans from 2-Hydroxyphenyl Acetylenes
2875	Copper[I]-catalyzed azide-alkyne cycloaddition
6003	Buchwald-Hartwig Ether Formation
6004	Suzuki-Miyaura Cross-Coupling (Bromo)
6005	Suzuki-Miyaura Cross-Coupling (Iodo)
6006	Suzuki-Miyaura Cross-Coupling (Chloro)
6008	Suzuki-Miyaura Cross-Coupling with Alkene
6009	Suzuki-Miyaura Cross-Coupling of Alkenes
6013	Hiyama Aryl-Alkenyl Cross-Coupling
6014	Hiyama Non-Aromatic Cross-Coupling
6015	Hiyama Allyl Cross-Coupling
6016	Hiyama Carbonylative Cross-Coupling
6017	Hiyama Cross-Coupling with Arylhydrazine
6022	Liebeskind-Srogl Thioamide Coupling
6024	Liebeskind-Srogl Nitrile Formation
6025	Liebeskind-Srogl Heterocyclic Coupling
6026	Sulfonamide Schotten-Baumann
6027	Sulfonamide Schotten-Baumann from Sulfonate
6028	Sulfonamide Schotten-Baumann from Thiol
6029	Sulfonamide Schotten-Baumann from Aryl Bromide
6031	Mitsunobu Reaction
6032	Mitsunobu carbon-carbon bond formation
6033	Mitsunobu SN2' Reaction
6034	Mitsunobu Imide Reaction
6035	Mitsunobu Aryl Ether Formation
6036	Mitsunobu Sulfonamide Reaction
6038	Ester or Amide or Thiolester Formation
6039	Williamson Ether Synthesis
6041	Buchwald-Hartwig Reaction - Amines
6043	Buchwald-Hartwig Reaction - Sulfonamides
7005	Benzimidazoles from o-Phenylenediamines
7009	Acylsulfonamide from Sulfonamide and Carboxylic Acid
7013	Benzimidazoles from o-Phenylenediamines and Aldehydes - Iodine
7014	Benzimidazoles from o-Phenylenediamines and Aldehydes - Boronic Acid
7015	Sulfonamide from sulfonic acid and amine
7017	Sulfonamide alkylation with a cyclic ether
7018	Sulfonamide acylation
7019	Wittig Reaction
7020	Wittig via Methoxy-Ylide
7021	Horner-Wadsworth-Emmons Olefination
7022	Chan-Lam coupling

Table S1: Names of the transforms.

ID	SAVI-Lib-2020 Pairs	SAVI-Lib-2020	SAVI-Space-2020 (Lib-2020 rules) Pairs	SAVI-Space-2020 (Lib-2020 rules)	SAVI-Lib-2020 products found in SAVI-Space-2020 (Lib-2020 rules) [%]
1031	153268	65568	444234	521864	99.90
1039	9185	1437	3621	9222	81.86
1171	464600	91508	442194	462257	94.38
1391	132464	20	113595	113940	100.00
1439	55803	42275	1504804	1530109	100.00
2201	3762026	582318	1164834	2662506	97.10
2218	7089	4376	5580	7286	97.66
2230	92950	45836	90035	92840	100.00
2238	18450355	1688760	27492084	28722114	98.00
2267	27406800	24239609	28436978	29131094	100.00
2269	201250	146610	472010	501683	97.56
2630	71288	10184	45630	45630	99.70
2684	3768	942	939	939	100.00
2875	1580160	1208372	1151172	1172488	99.50
6003	96702846	43731278	43582218	108609620	95.20
6004	5895351	5803732	6057577	6159830	95.30
6005	809070	804723	852475	852475	90.00
6006	7003410	2971508	5565480	6559756	83.20
6008	49413	49318	53664	53760	100.00
6009	453200	451616	470276	471789	96.60
6013	2982	2966	3052	3052	100.00
6014	11476	8976	9522	12087	100.00
6015	160	148	154	162	100.00
6016	24078	24052	25140	25146	100.00
6017	1106	1106	1114	1114	100.00
6022	89052	46698	92960	92960	55.60
6024	583	541	568	600	100.00
6025	177870	116790	176297	186186	99.60
6026	124375104	124373086	119264292	119264292	96.60
6027	6803352	6803243	6690168	6690168	93.40
6028	144541683	91702931	126795537	170806276	83.80
6029	423905867	211941152	415779650	415779650	93.70
6031	168433980	155748444	152036584	175226740	98.90
6032	213444	181524	181332	199800	99.00
6033	89016	83940	81909	87528	98.80
6034	37324658	27177967	22102226	29666259	62.90
6035	54868088	42306237	70447104	100927071	87.00
6036	21975210	5414034	11299840	21016589	99.90
6038	805366144	366277633	407093200	989263240	95.80
6039	345068989	103177836	317390470	317390470	95.90
6041	651821560	264493246	276087600	674877307	99.10
6043	37336404	32762479	33112170	37721992	99.90
7005	5635210	932282	957237	5630968	89.90
7009	49868520	46207962	46143390	49741994	99.60
7013	1761832	1151325	1415983	1753874	95.80
7014	723064	464185	582041	719798	97.10
7015	6802164	4856760	6026262	10412472	99.90
7017	11062500	3732596	7908600	11608110	99.30
7018	1234020	300300	611511	1249183	95.50
7019	494425960	142519769	166587415	291368239	93.10
7020	170100	11557	154310	183560	97.70
7021	35490	31843	31234	35182	97.90
7022	30682211	26185651	26404105	29818731	97.90

Table S2: Number of products for each transform for the SAVI-Lib-2020 and SAVI-Space-2020(Lib-2020 rules). The number of products before (pairs) and after the application of the rules is shown. Additionally the percantage of a testset of max. 1000 products found in the SAVI-Space-2020 is shown.

ID	r1	r2	pairs	r1 (w/o killed)	r2(w/o killed)	products (w/o killed)
1031	202376	192	9714048	201736	192	9683328
1039	26	83	2158	25	74	1850
1171	1166	1219	710677	5224	4548	691181
1439	740	8264	2038102	723	8264	1991284
2201	30954	888	8856717	10151	1498	2916466
2218	35445	5691	67239165	26235	4503	39378735
2230	144	5510	396720	142	5448	386808
2238	2379	47789	56842513	2309	95766	55206408
2267	7025	35793	117288042	6933	35512	115007370
2269	0	0	0	22848	48	1096704
2630	249	9724	2421276	249	9724	2421276
2684	188	1976	92872	188	1976	92872
2875	1950	3750	7312500	1935	3743	7242705
6003	24836	12756	316808016	17401	8651	150536051
6004	23308	1169	27247052	22769	1169	26616961
6005	3838	1200	4605600	3838	1200	4605600
6006	24102	1032	24873264	20544	999	20523456
6008	1201	120	144120	1200	120	144000
6013	3839	7	26873	3839	7	26873
6014	160	440	35200	131	396	25938
6015	3	88	264	3	82	246
6016	23926	2	47852	23914	2	47828
6017	853	2	1706	853	2	1706
6022	100	1201	120100	100	1201	120100
6024	1	1288	1288	1	1226	1226
6025	584	1169	682696	584	1113	649992
6026	2598	102615	266593770	2598	102615	266593770
6027	102862	198	20366676	102862	198	20366676
6028	102156	3129	319646124	92850	3353	311326050
6029	95026	14895	1415412270	95026	14895	1415412270
6031	46713	18086	844851318	45355	17674	801604270
6032	19246	22	423412	17789	22	391358
6033	48266	3	144798	45190	3	135570
6034	10910	38397	208133384	9079	35943	163160001
6035	19168	8835	169349280	17998	7754	139556492
6036	16652	3190	53119880	15521	2068	32097428
6038	85511	36906	3155868966	53408	30296	1618048768
6039	22247	35699	794195653	22247	35699	794195653
6041	60087	57124	1549655269	24418	27514	671836852
6043	22933	5023	115192459	21990	4538	99790620
7005	593	48255	28615215	201	23368	4696968
7009	2685	48046	129003510	2500	48058	120145000
7013	276	9725	2684100	249	8645	2152605
7014	418	9725	4065050	355	8688	3084240
7015	102815	198	20357370	79973	218	17434114
7017	2278	13304	30306512	1860	11561	21503460
7018	5974	493	2945182	3243	487	1579341
7019	18552	261951	748606973	14712	210532	474266184
7020	3	23978	71934	3	19724	59172
7021	9664	8	77312	8270	8	66160
7022	1095	267903	146599040	1060	240860	127645700

Table S3: Number of products for each transform of SAVI-Space-2024. The number of reactants and products before (pairs) and after the application of the kill statements is shown.

ID	r1	r2	pairs	r1 (w/o killed)	r2(w/o killed)	products (w/o killed)
1031	118732	176	5224208	118324	176	5206256
1039	18	57	1026	18	55	990
1171	972	784	381024	3865	2863	366294
1439	459	4992	763776	444	4992	738816
2201	17849	621	3623537	6216	1043	1264240
2218	21291	2556	18139932	16221	2118	11452026
2230	110	3328	183040	110	3270	179850
2238	1207	27378	16522272	1163	54862	15936009
2267	2358	19568	22268220	2315	19421	21711482
2269	0	0	0	13106	26	340756
2630	136	4898	666128	136	4898	666128
2684	52	1240	16120	52	1240	16120
2875	870	1220	1061400	858	1217	1044186
6003	13670	6272	85738240	9515	4267	40600505
6004	11050	529	5845450	10889	529	5760281
6005	1475	547	806825	1475	547	806825
6006	13505	464	6266320	11960	445	5322200
6008	548	68	37264	547	68	37196
6013	1476	2	2952	1476	2	2952
6014	78	230	8970	68	200	6800
6015	2	35	70	2	32	64
6016	11450	2	22900	11447	2	22894
6017	567	2	1134	567	2	1134
6022	75	548	41100	75	548	41100
6024	1	586	586	1	555	555
6025	296	535	158360	296	506	149776
6026	1850	59507	110087950	1850	59507	110087950
6027	59724	106	6330744	59724	106	6330744
6028	59244	2167	128381748	53414	2240	119647360
6029	55942	6934	387901828	55942	6934	387901828
6031	26731	9902	264690362	25983	9685	251645355
6032	10547	14	147658	9762	14	136668
6033	27508	3	82524	25935	3	77805
6034	6127	21008	63927123	5007	19658	49213803
6035	10499	4762	49996238	9858	4127	40683966
6036	9150	2144	19617600	8584	1270	10901680
6038	49019	21214	1039889066	31767	18004	571933068
6039	12281	20458	251244698	12281	20458	251244698
6041	32465	33347	481380085	13247	16652	220589044
6043	10818	3314	35850852	10362	3039	31490118
7005	369	27498	10146762	129	13808	1781232
7009	1685	27356	46094860	1562	27362	42739444
7013	200	4898	979600	180	4405	792900
7014	264	4898	1293072	231	4442	1026102
7015	59713	106	6329578	47055	121	5693655
7017	1436	7188	10321968	1180	6148	7254640
7018	3902	310	1209620	1969	306	602514
7019	10853	146851	229857063	8988	118840	154193229
7020	3	13732	41196	4	11236	44944
7021	4855	5	24275	4307	5	21535
7022	508	154796	39304846	500	138547	34635049

Table S4: Number of products for each transform of SAVI-Space-2020. The number of reactants and products before (pairs) and after the application of the kill statements is shown.

ID	r1	r2	pairs	r1 (w/o killed)	r2(w/o killed)	products (w/o killed)
1031	37276	14	521864	31731	14	444234
1039	174	53	9222	71	51	3621
1171	503	919	462257	503	882	442194
1391	15	7596	113940	15	7573	113595
1439	907	1687	1530109	892	1687	1504804
2201	13447	198	2662506	5883	198	1164834
2218	7286	1	7286	5580	1	5580
2230	55	1688	92840	55	1637	90035
2238	2034	14121	28722114	1954	14121	27492084
2267	1297	23978	29131094	1274	23800	28436978
2269	13559	37	501683	13486	35	472010
2630	9	5070	45630	9	5070	45630
2684	3	313	939	3	313	939
2875	904	1297	1172488	891	1292	1151172
6003	15020	7231	108609620	9858	4421	43582218
6004	11365	542	6159830	11197	541	6057577
6005	1525	559	852475	1525	559	852475
6006	13781	476	6559756	12205	456	5565480
6008	560	96	53760	559	96	53664
6009	89	5301	471789	89	5284	470276
6013	1526	2	3052	1526	2	3052
6014	79	153	12087	69	138	9522
6015	2	81	162	2	77	154
6016	12573	2	25146	12570	2	25140
6017	557	2	1114	557	2	1114
6022	166	560	92960	166	560	92960
6024	1	600	600	1	568	568
6025	342	546	186186	341	517	176297
6026	1932	61731	119264292	1932	61731	119264292
6027	61946	108	6690168	61946	108	6690168
6028	61574	2774	170806276	55539	2283	126795537
6029	58151	7150	415779650	58151	7150	415779650
6031	29185	6004	175226740	27503	5528	152036584
6032	11100	18	199800	10074	18	181332
6033	29176	3	87528	27303	3	81909
6034	4893	6063	29666259	3862	5723	22102226
6035	20109	5019	100927071	17199	4096	70447104
6036	9601	2189	21016589	8828	1280	11299840
6038	38696	25565	989263240	21800	18674	407093200
6039	12890	24623	317390470	12890	24623	317390470
6041	23474	34770	674877307	20291	17364	276087600
6043	11134	3388	37721992	10647	3110	33112170
7005	193	29176	5630968	69	13873	957237
7009	1714	29021	49741994	1590	29021	46143390
7013	346	5069	1753874	311	4553	1415983
7014	142	5069	719798	127	4583	582041
7015	61979	168	10412472	48994	123	6026262
7017	1530	7587	11608110	1225	6456	7908600
7018	3991	313	1249183	1979	309	611511
7019	9420	61702	291368239	7087	51412	166587415
7020	13	14120	183560	13	11870	154310
7021	5026	7	35182	4462	7	31234
7022	514	58020	29818731	504	52396	26404105

Table S5: Number of products for each transform of SAVI-Space-2020(Lib-2020 rules). The number of reactants and products before (pairs) and after the application of the kill statements is shown.

CHMTRN/PATRAN	SMARTS
X	*
C	#6
O	#8
N	#7
S	#16
P	#15
B	B
F	F
CL	Cl
I	I
BR	Br
SE	Se
SI	Si
SN	Sn
NA	Na
K	K
ALH	AlH

Table S6: SMARTS translations of CHMTRN/PATRAN for atom.

CHMTRN/PATRAN	SMARTS
ACETAL	[#6](-[#8]-[#6])-[#8]-[#6]
ACETYLENE	[#6]#[#6]
ACID	[#6;!$([#6]-[#7]);!$([#6](-[#8])-[#8])](=O)-[O;H1,-]
ACID*HALIDE	[#6;!$([#6]-[#7,#8])](=O)-[Cl,Br,I]
ALCOHOL	[#6;$([#6]-[O;H1,-]);!$([#6](~O)-O)]
ALDEHYDE	[#6;!$([#6]-[#7,#8]);$([#6;H1](=O)[#6]),$([#6;H2]=O)]
ALLENE	[#6]=[#6]=[#6]
AMIDE*1	[#6;!$([#6]-[#8])!$([#6](-[#7])-[#7])](=O)-[N;H2]
AMIDE*2	[#6;!$([#6]-[#8])!$([#6](-[#7])-[#7])](=O)-[#7;H1]-[#6]
AMIDE*3	[#6;!$([#6]-[#8])!$([#6](-[#7])-[#7])](=O)-[#7](-[#6])-[#6]
AMIDZ	[#6]-[#7]-[#6;!$([#6]-[#8])!$([#6](-[#7])-[#7])]=O
AMINE*1	[#6]-[#7;H2;!$([#7]-[#6]=[#7,#8])]
AMINE*2	[#6]-[#7;H1;!$([#7]-[#6]=[#7,#8])]-[#6]
AMINE*3	[#6]-[#7;!$([#7]-[#6]=[#7,#8])](-[#6])-[#6]
AMINE*OXIDE	[#6]-[#7+](-[#8-])(-[#6])-[#6]
ANHYDRIDE	[#6](=O)-[#8]-C=O
AZIDE	[#6]-[#7]=[#7+]=[#7-]
AZIRIDINE	C1-N-C1
AZO	[#6]-N=N-[#6]
BROMIDE	[#6]-Br
C*SULFONATE	[#6]-S(=O)(=O)-[O;H1,-]
CARBAMATE*C	[#6](-N(-[#6])(-[#6]))(=O)-O-[#6]
CARBAMATE*H	[#6](=O)([N;H1])-O
CARBONIUM	[C+]
CHLORIDE	[#6]-Cl
CYANO	[#6]#N
DIAZO	[#6]=[N+]=[N-]
DISULFIDE	[#6]-S-S-[#6]
DITHIOACETAL	[#6;H1](-[S;D2])-[S;D2]
DITHIOKETAL	[#6](-[#6])(-[#6])(-[S;D2])-[S;D2]
ENAMINE	[#6](=[#6])-N(-[#6])-[#6]
ENOL*ETHER	[#6](=[#6])-O-[#6]
ENOLIZABLE	[*;!H0;R0,R1;!$(*=,#,:*);$([*;$([#6](=O)-[Cl,Br,I]),<br>$([#6;$([#6;H1](=O)[#6]),$([#6;H2]=O)]),$([#6](=O)-[N;H2]),<br>$([#6](=O)-[#7;H1]-[#6]),$([#6](=O)-[#7](-[#6])-[#6]),<br>$([#6](=O)-[#8]-C=O),$([C+]),$([#6](=O)-[#8]-[#6]),<br>$([#6](-[#6])(=O)-[#6]),$([#6]=[#7$([#7;H1]),$([#7]-[#6])]),<br>$([#6]#N),$([#6]=N-[O;H1]),$([#6](=O)-[#16]-[#6]),<br>$([#6](-[F,Cl,Br,I])(-[F,Cl,Br,I])-[F,Cl,Br,I]),<br>$([#6]-[N+](=O)-[O-]),$([#6]-S(=O)(=O)-[O;H1]),<br>$([#6]-S(=O)(=O)-[#6]),$([#6]-[#16](=O)-[#6])]),<br>$(*~[*;$([#6](=O)-[Cl,Br,I]),<br>$([#6;$([#6;H1](=O)[#6]),$([#6;H2]=O)]),<br>$([#6](=O)-[N;H2]),$([#6](=O)-[#7;H1]-[#6]),<br>$([#6](=O)-[#7](-[#6])-[#6]),$([#6](=O)-[#8]-C=O),<br>$([C+]),$([#6](=O)-[#8]-[#6]),$([#6](-[#6])(=O)-[#6]),<br>$([#6]=[#7$([#7;H1]),$([#7]-[#6])]),$([#6]#N),<br>$([#6]=N-[O;H1]),$([#6](=O)-[#16]-[#6]),<br>$([#6](-[F,Cl,Br,I])(-[F,Cl,Br,I])-[F,Cl,Br,I])])]

Table S7: SMARTS translations of CHMTRN/PATRAN for functional groups. (Part 1)

CHMTRN/PATRAN	SMARTS
EPISULFIDE	[#6]1-S-[#6]1
EPOXIDE	[#6]1-O-[#6]1
ESTER	[#6;!$([#6]-[#7])!$([#6](-[#8])-[#8])](=O)-[#8]-[#6]
ESTERX	[#6]-[#8]-[#6;!$([#6]-[#7])!$([#6](-[#8])-[#8])]=O
ETHER	[#6;$([#6]-[#8]-[#6]);!$([#6](-[#8])-[#8]-[#6]);<br>!$([#6](=O)-[#8]-[#6]);!$([#6]-[#8]-[#6]=O)]
FLUORIDE	[#6]-F
GEM*DIHALIDE	[#6](-[F,Cl,Br,I])-[F,Cl,Br,I]
GLYCOL	[#6](-[O$([O;H1]),$(O-[#6])])-[#6]-[O$([O;H1]),$(O-[#6])]
HALIDE	[#6]-[F,Cl,Br,I]
HALOAMINE	[#6]-[#7]-[F,Cl,Br,I]
HALOHYDRIN	[#6](-[F,Cl,Br,I])-[O$([O;H]),$(O-[#6])]
HEMIACETAL	[#6;H1](-[#8]-[#6])-[O;H1]
HYDRATE	[#6](-[O;H1])-[O;H1]
HYDRAZONE	[#6]=N-N
HYDROXYLAMINE	[#6]-[#7]-[O;H1]
IMINE	[#6]=[#7$([#7;H1]),$([#7]-[#6])]
IODIDE	[#6]-I
ISOCYANATE	[#6]-N=[#6]=O
ISOCYANIDE	[#6]-[#7+]#[C-]
KETONE	[#6](-[#6])(=O)-[#6]
LACTAM	[#6;R](=O)@[N;H1;R]@[#6;R]
LACTONE	[#6;R](=O)@[O;R]@[#6;R]
METHYLENE	[#6]=[#6;H2]
N*CARBAMATE	[#6]-N-[#6](=O)-O-[#6]
N*UREA*C	[#6]-N(-[#6])-[#6](=O)-N(-[#6])-[#6]
N*UREA*H	[#6;$([#6]-N-[#6](=O)-[N;H1]),$([#6]-[N;H1]-[#6](=O)-N)]
NITRILE	[#6]#N
NITRITE	[#6]-O-N=O
NITRO	[#6]-[N+](=O)-[O-]
NITROSO	[#6]-N=O
O*CARBAMATE	[#6]-O-[#6](=O)-N
O*CARBONATE	[#6]-O-[#6](=O)-O-[#6]
O*SULFONATE	[#6]-O-[S$(S-[#6]),$([S;H1])](=O)(=O)
OLEFIN	C=C
OXIME	[#6]=N-[O;H1]
PHENYL	c1[cH][cH][cH][cH][cH]1
PEROXIDE	[#6]-O-[O$([O;H1]),$(O-[#6])]
PHOSPHINE	[#6]-P(-[#6])-[#6]
PHOSPHONATE	[#6]-O-P
SELENIDE	[#6]-[Se]-[#6]
SILYLENOLETHER	[#6](=C)-O-[Si](-[#6])(-[#6])-[#6]
SULFIDE	[#6]-[#16]-[#6]
SULFONE	[#6]-S(=O)(=O)-[#6]
SULFOXIDE	[#6]-[#16](=O)-[#6]
THIOCYANATE	[#6]-S-C#N
THIOESTER	[#6](=O)-[#16]-[#6]
THIOL	[#6]-[S;H1]
T*BUTYL	[#6]-C(-C)(-C)-C
TRIALKYLSILOXY	[#6]-O-[Si](-[#6])(-[#6])-[#6]
TRIALKYLSILYL	[#6]-[Si](-[#6])(-[#6])-[#6]
TRIHALIDE	[#6](-[F,Cl,Br,I])(-[F,Cl,Br,I])-[F,Cl,Br,I]
VIC*DIHALIDE	[#6](-[F,Cl,Br,I])-[#6]-[F,Cl,Br,I]
VINYLSILANE	[#6](=C)-[Si](-[#6])(-[#6])-[#6]

Table S8: SMARTS translations of CHMTRN/PATRAN for functional groups. (Part 2)

CHMTRN/PATRAN	Synonym
SULPHIDE	SULFIDE
SULPHONE	SULFONE
SULPHOXIDE	SULFOXIDE
O*SULPHONATE	O*SULFONATE
NITRILE	CYANO
HYDROXYL	ALCOHOL
EPISULPHIDE	EPISULFIDE
DISULPHIDE	DISULFIDE
C*SULPHONATE	C*SULFONATE
CARBONYL	ALDEHYDE, KETONE
CARBOXYL	ACID, ESTER, AMIDE*1, AMIDE*2, AMIDE*3, ACID*HALIDE, THIOESTER, ANHYDRIDE
AMINE	AMINE*1, AMINE*2, AMINE*3
AMIDE	AMIDE*1, AMIDE*2, AMIDE*3
LEAVING	ACETAL, ALCOHOL, AMIDZ, AZIRIDINE, O*CARBAMATE, N*CARBAMATE, DITHIOACETAL, DITHIOKETAL, EPISULFIDE, EPOXIDE, ESTERX, ETHER, GEM*DIHALIDE, HALIDE, N*UREA*C, N*UREA*H, O*CARBONATE, PHOSPHONATE, SELENIDE, SULFIDE, SULFONE, C*SULFONATE, O*SULFONATE, THIOCYANATE, THIOL, TRIALKYLSILOXY, TRIHALIDE
GOOD*LEAVING	AZIRIDINE, BROMIDE, CHLORIDE, EPISULFIDE, EPOXIDE, IODIDE, O*SULFONATE, PHOSPHONATE, GEM*DIHALIDE, TRIHALIDE
XWITHDRAWING	ACID*HALIDE, ALDEHYDE, AMIDE*1, AMIDE*2, AMIDE*3, ANHYDRIDE, CARBONIUM, ESTER, KETONE, IMINE, NITRILE, OXIME, THIOESTER, TRIHALIDE
NONEXPANDABLE*WITHDRAWING	NITRO, C*SULFONATE, SULFONE, SULFOXIDE
WITHDRAWING	ACID*HALIDE, ALDEHYDE, AMIDE*1, AMIDE*2, AMIDE*3, ANHYDRIDE, CARBONIUM, ESTER, KETONE, IMINE, NITRILE, OXIME, THIOESTER, TRIHALIDE, NITRO, C*SULFONATE, SULFONE, SULFOXIDE
DONATING	ACETAL, ALCOHOL, AMIDZ, AMINE*1, AMINE*2, AMINE*3, ESTERX, ETHER, HALOAMINE, HEMIACETAL, HYDROXYLAMINE, N*CARBAMATE, N*UREA*C, N*UREA*H, O*CARBAMATE, O*CARBONATE, PHOSPHINE, SELENIDE, SULFIDE, THIOL, TRIALKYLSILOXY

Table S9: SMARTS translations of CHMTRN/PATRAN for synonym.

CHMTRN/PATRAN	SMARTS
DIARYL	a;R;$(*(@;:*)(@;:*)@;:*)
ALKYLARYL	R;$(*(@;:*)(@;:*)@;!:*),$(*(@;:*)(@;:*)@;:*@;!:*)
DIALKYL	A;R;$(*(@;!:*)(@;!:*)@;!:*)&!$(*(@;!:*)(@;!:*)(@;!:*)@;!:*),$(*(@;!:[*;!R1])@;!:[*;!R1])
TRIALKYL	A;R3;$(*(@;!:*)(@;!:*)(@;!:*)@;!:*)
YES	!R0;!R1;$(*(@*)(@*)@*)&!$(*(@*)(@*)(@*)@*),$(*(@[*;!R1])@[*;!R1]),R3&$(*(@*)(@*)(@*)@*)
NO	only for syntax check
EITHER	

Table S10: SMARTS translations of CHMTRN/PATRAN for fusion.

CHMTRN/PATRAN	SMARTS
DIARYL	!$(*(:*)(:*):*)
ALKYLARYL	!$(*(:*)(:*)@;!:*);!$(*(:*)(:*):*@;!:*)
DIALKYL	!$(*(@;!:*)(@;!:*)@;!:*),$(*(@;!:*)(@;!:*)(@;!:*)@;!:*);!$(*(@;!:[*;!R1])@;!:[*;!R1])
TRIALKYL	!$([A;R3])
YES	R0,R1,!$(*(@*)(@*)@*),$(*(@*)(@*)(@*)@*);!$(*(@[*;!R1])@[*;!R1]);!R3,!$(*(@*)(@*)(@*)@*)
NO	only for syntax check

Table S11: SMARTS translations of CHMTRN/PATRAN for no fusion.

CHMTRN/PATRAN	SMARTS
-	-
=	=
#	#
%	:
&	~
+	.

Table S12: SMARTS translations of CHMTRN/PATRAN for bond.

CHMTRN/PATRAN	SMARTS
ANION	-1,-2,-3,-4
CATION	+1,+2,+3,+4
NEUTRAL	
RADICAL	
YES	!+0
NO	+0
EITHER	

Table S13: SMARTS translations of CHMTRN/PATRAN for charge.

CHMTRN/PATRAN	SMARTS
ALPHA	~
BETA	~*~
GAMA	~*~*~

Table S14: SMARTS translations of CHMTRN/PATRAN for positional statements.
